# Population genetic diversity and natural *Wolbachia* infection in *Aedes aegypti* from Pakistan

**DOI:** 10.1186/s13071-025-07134-x

**Published:** 2025-11-24

**Authors:** Jehangir Khan, Datao Lin, Amer Al-Jawabreh, Abdul Aziz, Dongjing Zhang, Chen Tao, Han Qian

**Affiliations:** 1 Laboratory of Tropical Veterinary Medicine and Vector Biology, School of Life and Health Sciences, Hainan Province Key Laboratory of One Health, Collaborative Innovation Center of Life and Health, Hainan University, Haikou, 570228 Hainan China; 2 Hainan General Hospital, Hainan Affiliated Hospital of Hainan Medical University, Haikou, 570100 Hainan China; 3 Zoology Department, Abdul Wali Khan University, Mardan, Pakistan; 4 Chinese Atomic Energy Agency Center of Excellence On Nuclear Technology Applications for Insect Control, Key Laboratory of Tropical Disease Control of the Ministry of Education, Sun Yat-Sen University, Guangzhou, China; 5 Department of Parasitology, Key Laboratory of Tropical Disease Control (Ministry of Education), Zhongshan School of Medicine, Sun Yat-Sen University, Guangzhou, China; 6 Department of Medical Laboratory Sciences, Faculty of Allied Health Sciences, Arab American University, Jenin, Palestine; 7 Leishmaniases Research Unit, Jericho, Palestine; 8 Nuclear Institute for Food and Agriculture (NIFA), Peshawar, Khyber Pakhtunkhwa Pakistan; 9 Hainan Provincial Bureau of Disease Prevention and Control, Haikou, 570100 China

**Keywords:** *Aedes aegypti*, *COI* gene, Genetic diversity, Phylogeography, *Wolbachia*

## Abstract

**Background:**

*Aedes aegypti*, the principal vector of dengue and other arboviruses, is widely distributed in Pakistan, yet its population genetics and endosymbiont status remain poorly characterized. This study aimed to investigate the genetic structure, haplotype diversity, and phylogeographic patterns of *Ae. aegypti* in dengue-endemic regions of Pakistan, and to screen for natural *Wolbachia* infections to provide baseline data for surveillance and vector control.

**Methods:**

Ovitrap collections were conducted in 2021 across the provinces of Punjab (Bakkar) and Khyber Pakhtunkhwa (Charsadda, DI Khan, Kohat, and two sites within Peshawar: Hayat Abad and Tarnab). Following the morphological identification of adult *Ae. aegypti*, we extracted genomic DNA from confirmed specimens to amplify and sequence a 658-bp fragment of the mitochondrial cytochrome c oxidase I (*COI*) gene. Phylogenetic analyses, haplotype network construction, and population differentiation statistics were performed. Additionally, 300 field-caught adult mosquitoes were screened for *Wolbachia* using validated conventional and quantitative PCR assays targeting the *Wolbachia* surface protein (*wsp*) gene.

**Results:**

Phylogenetic analysis of 166 COI sequences (92 from Pakistan) revealed a monophyletic *Ae. aegypti* clade with 99.65–100% sequence identity, with Pakistani isolates clustering with those from Saudi Arabia, Iran, and India. In total, 13 global haplotypes were identified, with Hap_3 dominating (53%) and shared across regions. Within Pakistan, eight haplotypes were detected, including region-specific variants, yielding high overall diversity (Hd 0.69; *π* = 0.007). District-level analysis showed that DI Khan and Bakkar had the highest haplotype diversity (Hd 0.73 and 0.71) but low nucleotide diversity (*π* = 0.005–0.006), whereas Kohat exhibited no haplotype diversity. Population structure was higher in Pakistan (FST 0.26; Nm 0.7) than globally (FST 0.17; Nm 1.19), consistent with low gene flow among Pakistani populations. No natural *Wolbachia* infections were detected in *Ae. aegypti*.

**Conclusions:**

*Aedes aegypti* in Pakistan belong to a globally monophyletic lineage and show moderate mitochondrial diversity with higher population structure than the global population. The lack of detected *Wolbachia* infections suggests that natural strains are either absent or occur at very low prevalence. These findings provide a baseline for surveillance and support integrating *Wolbachia*-based biocontrol alongside conventional interventions in Pakistan.

**Graphical Abstract:**

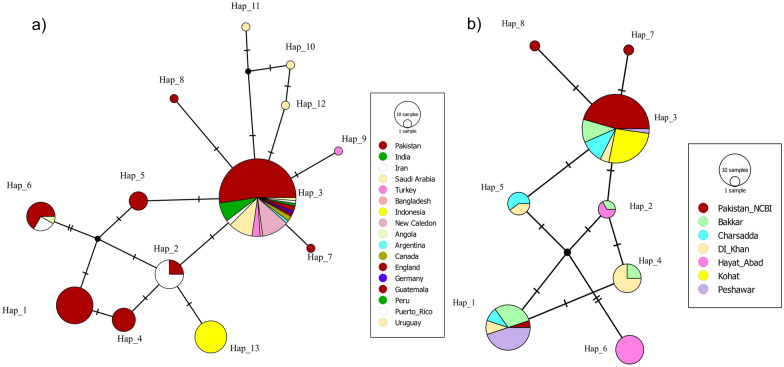

**Supplementary Information:**

The online version contains supplementary material available at 10.1186/s13071-025-07134-x.

## Background

*Aedes aegypti* is a highly invasive mosquito and the principal global vector of arboviruses, including dengue and Zika, which collectively threaten millions of people each year [1]. Its global spread is driven by ecological adaptability, anthropophilic behavior, and urban resilience, especially in tropical and subtropical regions. This expansion poses serious public health threats, particularly in South and Southeast Asia [2, 3].

In Pakistan, dengue has become hyperendemic, with an estimated 286,262 reported cases and 1108 deaths from major outbreaks [4]. *Aedes aegypti* has been the primary vector driving these recurrent epidemics since its first detection in the mid-twentieth century [5, 6]. While earlier studies [7, 8] have provided initial insights into the genetics of Pakistani mosquito populations, key aspects such as the fine-scale spatial genetic structure, evolutionary history, and potential for local adaptation of *Ae. aegypti* remain poorly resolved. This knowledge gap limits the ability to model dispersal patterns or develop localized vector control strategies [9, 10].

Mitochondrial markers, particularly the cytochrome c oxidase subunit I (*COI*) gene, are widely used in mosquito phylogeography owing to their maternal inheritance and mutation rate, which are effective for tracing lineages and assessing population connectivity [8, 11–14]. However, a critical consideration for interpreting mitochondrial DNA (mtDNA) data is the potential confounding effect of the maternally inherited endosymbiotic bacterium *Wolbachia*. As both elements are co-inherited, *Wolbachia*-induced selective sweeps can distort mitochondrial haplotype frequencies, reduce genetic diversity, and create patterns that misleadingly suggest recent population expansion or gene flow, as demonstrated in *Ae. albopictus* and other insects [15–18]. Therefore, *Wolbachia* screening is essential to validate mtDNA-based inferences and ensure accurate phylogeographic reconstruction.

Beyond its relevance for population genetics, establishing the *Wolbachia* infection status of wild populations is crucial for assessing the feasibility of novel biocontrol strategies. The artificial introduction of *Wolbachia* into *Ae. aegypti* populations has been successfully deployed to suppress arbovirus transmission [19–21]. Although natural *Wolbachia* infections in *Ae. aegypti* are considered rare or absent [21], sporadic reports from neighboring India [22, 23] and other regions [24–27] underscore the critical need for country-specific screening to validate mtDNA-based inferences and assess biocontrol readiness.

Therefore, we present here an integrated study of *Ae. aegypti* populations from ecologically diverse districts in Pakistan, combining mitochondrial COI sequencing with rigorous *Wolbachia* screening. Specifically, we aimed to (1) characterize the genetic diversity, population structure, and phylogeographic patterns of *Ae. aegypti* in Pakistan, and (2) determine the prevalence of natural *Wolbachia* infections. This dual approach enables a robust interpretation of the genetic data while providing a critical baseline for future biocontrol assessments, offering a comprehensive foundation for evidence-based vector management in Pakistan.

## Methods

### Study area

**Fig. 1:**
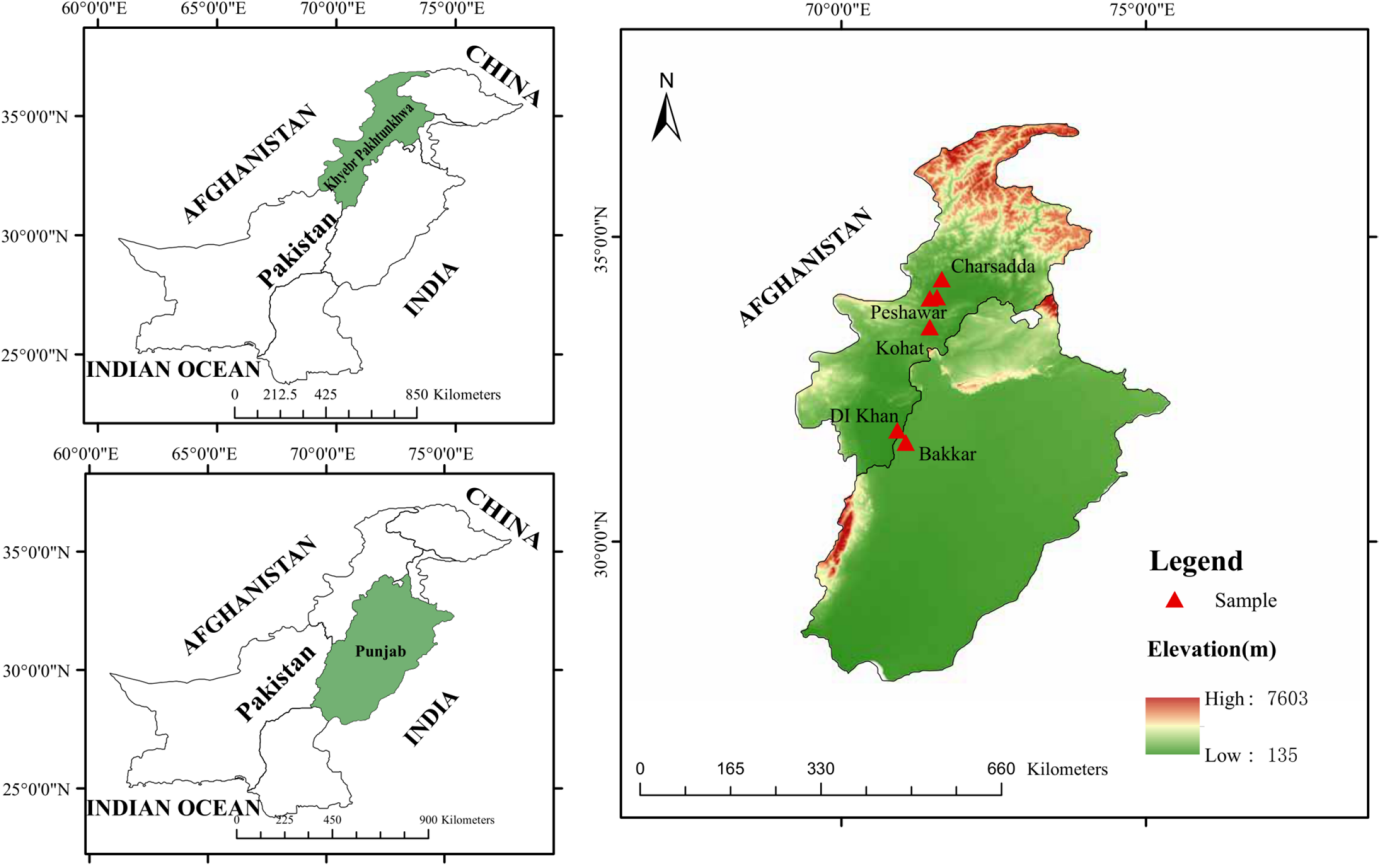
Geographic distribution of *Ae. aegypti* sampling sites in Pakistan. Hayat Abad and Tarnab are located within Peshawar District. The map was generated in ArcGIS 10.5 using open-access base layers from Natural Earth. Site coordinates were obtained in the field with GPS and are presented in Table 1

Mosquito sampling was conducted across six geographic locations across five dengue-endemic districts in Pakistan [4]: Peshawar, Charsadda, Kohat, Dera Ismail Khan (DI Khan) in KP, and Bakkar in Punjab (Fig. 1). These sites were selected to represent diverse ecological habitats, from urban areas to rural and semi-arid zones, enabling a comprehensive analysis of *Ae. aegypti* populations [28]. In Peshawar, collections were carried out at Tarnab, a rural agricultural interface, and Hayat Abad, a densely populated urban neighborhood. The Charsadda sampling site (Tangi) represented a rural, irrigated setting embedded within an agricultural landscape. Kohat, characterized by its semi-arid, upland terrain, offered a distinct ecological profile with reduced vegetation and more variable seasonal conditions. DI Khan, located along the Indus River in southern KP, is a known hotspot for dengue activity and features extensive canal networks and urban settlements favorable to mosquito breeding. Bakkar, in southwestern Punjab, lies at the edge of the Thal Desert and represents an arid, lowland district where urban expansion and water storage practices have facilitated recent *Ae. aegypti* establishment [29]. Table 1 provides climatic characteristics, geographic coordinates, and sampling metadata for all sites.

**Table 1:** Summary of sampling and collection sites, climatic conditions, and *COI* sequence data for *Ae. aegypti* Populations in Pakistan

Sampling site	Province	Latitude	Longitude	Elevation (m)	SM^a^	Isolates^b^	Accession no.	AMT^c^ (°C)	AMH^d^ (%)	Comments
Bakkar	Punjab	31.6277	71.0625	169	Ovitraps	B1-B14	PV925869-82	24.6	34	Hot and dry summers
Charsadda	KP^**e**^	34.3009	71.6524	301	Ovitraps	C1-C10	PV925883-92	22.5	59	Flat plains, agricultural surroundings
DI Khan	KP	31.8166	70.91667	177	Ovitraps	D1-D12	PV925893-904	25.3	59	High summer temps, low humidity
Kohat	KP	33.5225	71.4464	489	Ovitraps	K1-K12	PV925915-26	19.2	55	Semi-arid zone, higher elevation
Hayat Abad Peshawar	KP	33.9861	71.4569	331	Ovitraps	H1-H10	PV925905-14	22.0	60	Urban center, variable climate
Tarnab Peshawar	KP	34.0144	71.5675	331	Ovitraps	T1-T10	PV925927-36	22.0	60	Rural area, variable climate

^a^Sampling Method

^b^Sequences included in final analysis

^c^Annual Mean Temp

^d^Annual Mean Humidity

^e^Khyber Pakhtunkhwa

### Mosquito sampling and colony maintenance

Sampling was conducted during peak mosquito activity (July–December 2021) using 15–20 ovitraps spaced at 100 m intervals per site to ensure comprehensive coverage. This sampling strategy was optimized for broad spatial coverage to capture population-level genetic diversity and has been previously validated for efficient *Ae. aegypti* collection in the local ecological context [28]. Eggs and larvae were collected bi-monthly and transported to the Nuclear Institute for Food and Agriculture (NIFA), Peshawar, and the Zoology Department, Abdul Wali Khan University Mardan (AWKUM), for rearing under standard laboratory conditions (28 ± 2 °C, 70 ± 5% RH, 12:12 L:D photoperiod) [30]. The subsequently emerged adults were identified morphologically using standard keys [31] and prepared for molecular analysis.

### DNA extraction

Genomic DNA was extracted from individual mosquitoes using the DNeasy Blood and Tissue Kit (Qiagen, Hilden, Germany). Overall, 50 adult mosquito isolates were randomly collected and processed from each district. Each specimen was homogenized in 500 µL STE buffer using a sterile steel bead at 50 Hz for 30–60 s [30]. DNA concentration and purity were assessed using a NanoDrop spectrophotometer (Thermo Scientific, USA). Extracts with concentrations of 30–50 ng/µL and A260/280 ratios between 1.8 and 2.0 were deemed acceptable. DNA integrity was further verified by 1% agarose gel electrophoresis. On the basis of these quantifications, approximately 60–100 ng of genomic DNA was used as a template for each PCR reaction to ensure standardized input. Only high-quality DNA was used for downstream COI amplification and *Wolbachia* detection assays. Extracted DNA was stored at −20 °C or used immediately.

### PCR amplification of the cytochrome oxidase subunit I gene

A 658-bp fragment of the *COI* gene was amplified using the universal primers LCO1490 and HCO2198 [32]. Each 25 μL PCR reaction contained 12.5 μL of 2 × Taq PCR Master Mix (Thermo Scientific), 0.5 μM of each primer, and 2 μL of genomic DNA. The thermocycling protocol consisted of an initial denaturation at 94 °C for 3 min; 35 cycles of 94 °C for 30 s, 50 °C for 30 s, and 72 °C for 1 min; followed by a final extension at 72 °C for 10 min. PCR products were visualized on 1.5% agarose gels stained with ethidium bromide. The PCR products with successful amplicons were sent for bidirectional Sanger sequencing (Sangon Biotech, Guangzhou, China).

### *Wolbachia* screening via conventional and quantitative PCR

To assess natural *Wolbachia* infection status while avoiding artifacts introduced by laboratory colonization, we screened individual field-caught adult *Ae. aegypti* (*n* = 300; 50/district) from multiple districts using PCR targeting the wsp gene [33], with laboratory-specific optimizations from our previous study [34]. This approach prevents infection loss during laboratory colonization and complements larval collections that reflect site-specific breeding populations. Initial screening was performed using conventional PCR with primers 81 F and 691R (−600 bp amplicon) under standardized cycling conditions [34]. Amplicons of the expected size were visualized by agarose gel electrophoresis. To verify positives and estimate infection density, a short-amplicon quantitative PCR (qPCR) assay (wsp, −150 bp) was employed following established methods [34]. qPCR reactions (20 μL) employed SYBR Green Master Mix and the same primer pair under the following cycling conditions: 95 °C for 10 min; 40 cycles of 95 °C for 15 s and 60 °C for 1 min; followed by a melt curve analysis (60–95 °C with a 0.3 °C/s ramp). Each sample was additionally amplified for an endogenous *Ae. aegypti* gene (rps17, −150 bp) as an internal control to confirm DNA integrity and rule out PCR inhibition. Samples were deemed *Wolbachia*-positive only if they met all of the following criteria: (i) quantification cycle (Cq) ≤ 35, (ii) a single-peak dissociation (melt) curve, and (iii) electrophoretic confirmation of the expected amplicon size. Each run included stringent controls: *Wolbachia*-positive DNA from *Ae. albopictus* and *Culex pipiens* (positive controls), nuclease-free water (no-template negative control), and extraction blanks. Positive controls consistently amplified with Cq values between 17.5 and 21.0, confirming assay sensitivity.

### Population genetic analyses

Raw *COI* gene sequences were processed using BioEdit v7.2.5 for trimming and cleaning, with chromatogram verification in Chromas v2.31 (www.technelysium.com.au/chromas.html). Homologous sequences were retrieved from GenBank using BLAST v2.14.0 [35], and multiple sequence alignment (99.98–100%) was conducted in MEGA v12 (ClustalW) [36]. Poor-quality sequences (e.g., ambiguous bases or weak signals) were excluded. From 50 adults processed per district, 20 were sequenced, and 10–14 high-quality sequences were retained per site (Table 1). The final dataset (*n* = 166) comprised 68 new sequences, 24 archived Pakistani sequences [8], and 74 global references (Additional file 1: Table S1), all trimmed to uniform length for downstream analyses.

Phylogenetic relationships [37] were inferred using IQ-TREE v2.1.4, with substitution models selected via ModelFinder (TIM3 + F + I + G4 for global and TN + F + I + G4 for Pakistani datasets). Node support was assessed using 1000 ultrafast bootstraps and SH-like aLRT tests [38, 39]. Outgroups included *Anopheles gambiae*, *An. funestus*, and *An. arabiensis*. Trees were visualized in FigTree v1.4.4.

Genetic diversity indices (S, h, Hd, π) and neutrality tests (Tajima’s D, Fu’s Fs) were calculated in DnaSP v6.12.03 [40], while the haplotype network was generated using the Templeton, Crandall, and Sing (TCS) statistical parsimony algorithm implemented in PopArt v1.7 [41]. This analysis facilitated the visualization of haplotype connectivity and geographic distribution patterns [41].

We further assessed the spatial genetic structure of *Ae. aegypti* within Pakistan on the basis of *COI* sequence variation to detect fine-scale patterns potentially obscured in global-scale phylogenies. The dataset included two groups: (i) previously published Pakistani sequences from GenBank (“Pakistan_NCBI”) and (ii) 68 newly generated sequences from six districts. Population differentiation was quantified using pairwise FST values in Arlequin v3.5.2.2 [42] with 10,000 permutations and Bonferroni correction. Analysis of Molecular Variance (AMOVA) partitioned variance among groups, among populations, and within populations. Visualization of FST patterns was performed with heatmaps and hierarchical clustering in Clustvis (https://biit.cs.ut.ee/clustvis/) and ChiPlot (https://www.chiplot.online/). Additional clustering was evaluated using UPGMA based on Kimura 2-parameter (K2P) distance.

## Results

### Phylogenetic relationships of *Ae. aegypti* populations

**Fig. 2:**
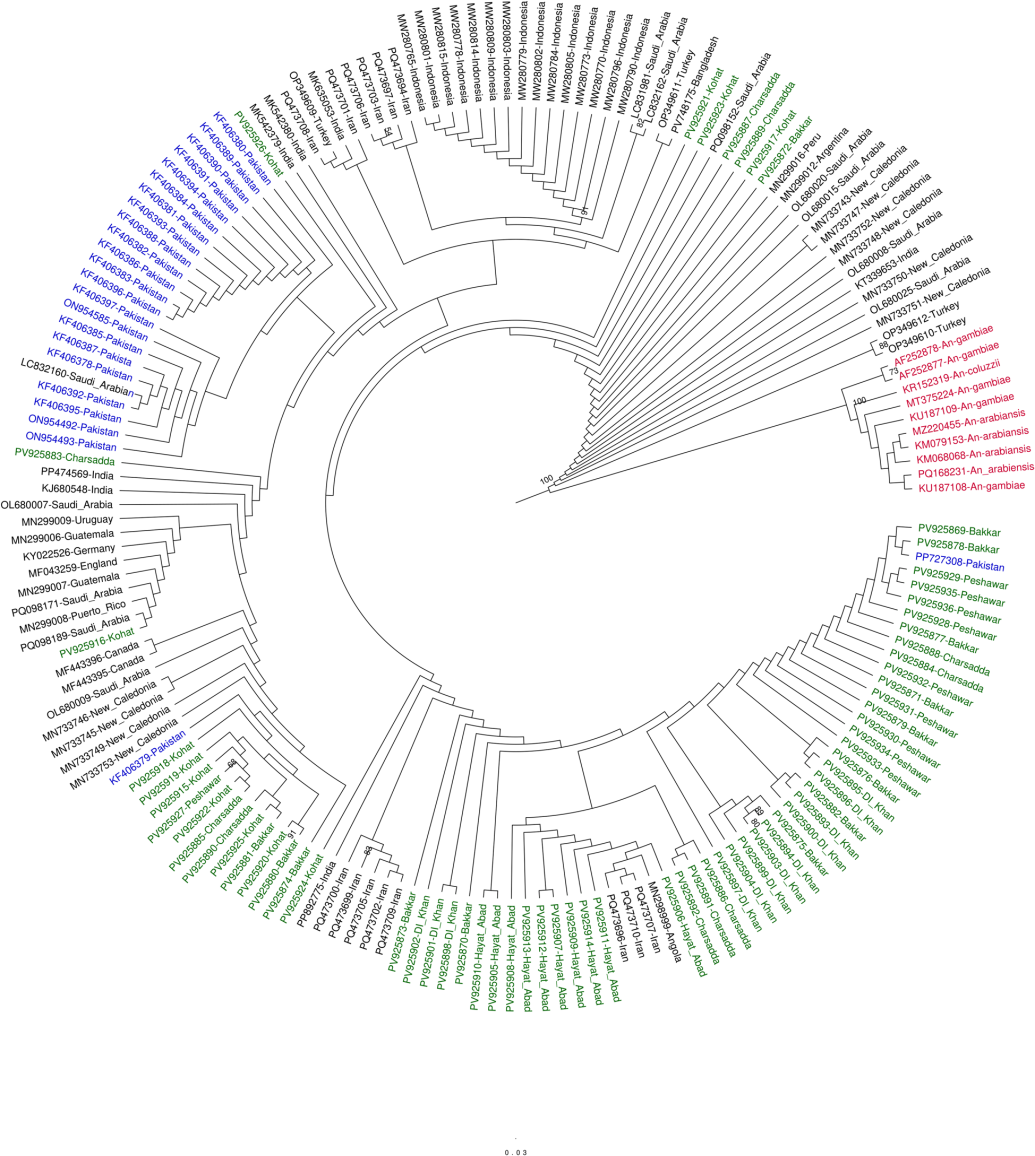
Maximum likelihood (ML) phylogeny reconstructed using 166 *Ae. aegypti COI* sequences, including 92 from Pakistan (newly generated sequences in green; NCBI-archived in blue) and 74 global reference strains. Bootstrap values ≥ 72 are shown at the nodes. The tree was rooted with outgroup taxa from related *Anopheles* species (marked in red), confirming the monophyly of *Ae. aegypti* with strong statistical support. Node stability was evaluated through 1000 bootstrap replicates (values indicating cluster recovery frequency) and Shimodaira-Hasegawa-like approximate likelihood ratio tests (SH-aLRT; 1000 replicates). Evolutionary divergence was calculated using the TIM3 + F + I + G4 substitution model, selected as optimal for this dataset. The scale bar indicates substitutions per site. Final tree visualization and annotation were performed in FigTree v1.4

The maximum likelihood (ML) phylogenetic tree constructed from 166 *COI* sequences revealed a well-supported monophyletic clade of *Ae. aegypti*, clearly separated from the *Anopheles* outgroup (Fig. 2). The newly generated sequences (*n* = 68) exhibited high identity (99.65–100%) with both previously submitted Pakistani (*n* = 24) [8] and global reference sequences (*n* = 74) (see Additional file 1: Table S1 for sequence details). Internal similarity among new sequences ranged from 99.89–100%, indicating high sequence conservation across isolates. Most Pakistani isolates clustered with sequences from geographically proximate regions, including Saudi Arabia (e.g., LC218160, KF406391), India (MH432759), and Iran (e.g., PO473709, PO473710), supported by short branch lengths and high bootstrap values (> 85%). In contrast, isolates from more geographically distant populations (e.g., Indonesia, New Caledonia, Uruguay, Germany, and Argentina) formed divergent clades. Previously submitted Pakistani sequences appeared in both overlapping and distinct lineages, highlighting intra-country variability and historical gene flow with surrounding regions.

**Fig. 3:**
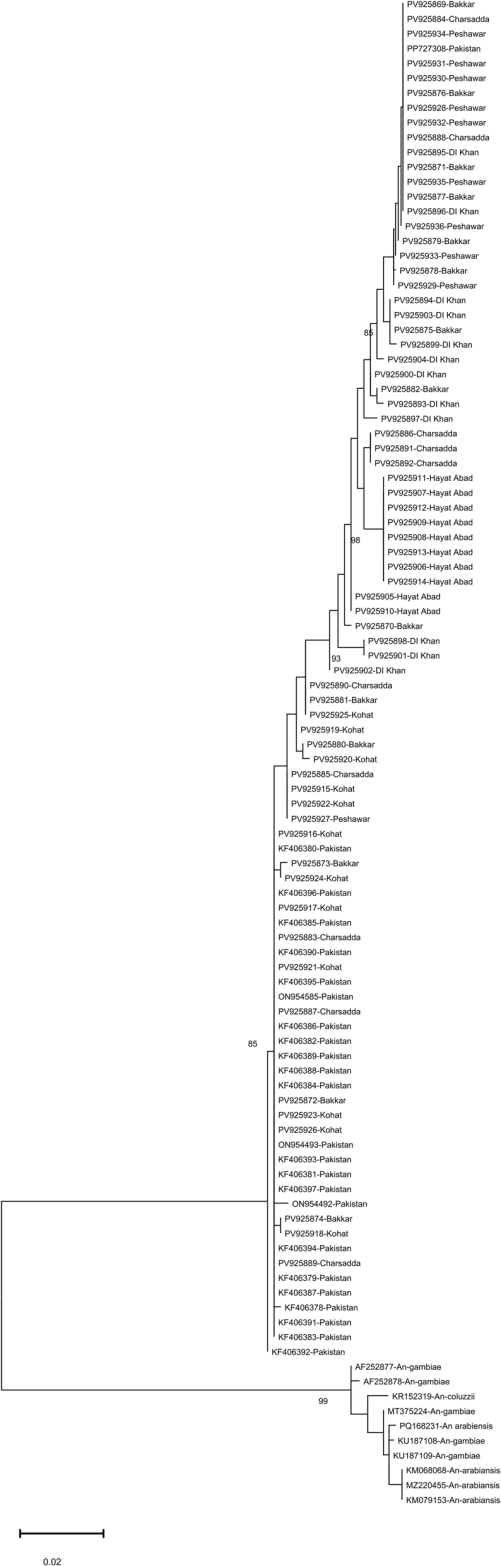
Maximum likelihood phylogeny of Pakistani *Ae. aegypti* populations based on 92 *COI* sequences (68 newly generated and 24 from NCBI), rooted with *Anopheles* spp. outgroups. Bootstrap values ≥ 72 are shown at the nodes. Node support values represent bootstrap percentages (1000 replicates) and Shimodaira-Hasegawa-like approximate likelihood ratio tests (SH-aLRT; 1000 replicates). Evolutionary distances were computed under the TN + F + I + G4 nucleotide substitution model. The scale bar indicates substitutions per site. Tree visualization was performed using FigTree v1.4

To further assess fine-scale structuring, a separate ML tree was constructed using only Pakistani *COI* sequences (*n* = 92) (Fig. 3). All sequences formed a monophyletic clade, and sequences from different sites were intermixed across subclades, with only moderate bootstrap support (≥ 85) at several nodes. A single well-supported cluster (bootstrap = 98) comprised eight sequences from Hayat Abad (district Peshawar), reflecting low genetic variability due to localized sampling or limited gene flow. The overall topology was shallow, with short branch lengths and no consistent geographic structuring.

### Phylogeography and haplotype distribution

**Fig. 4:**
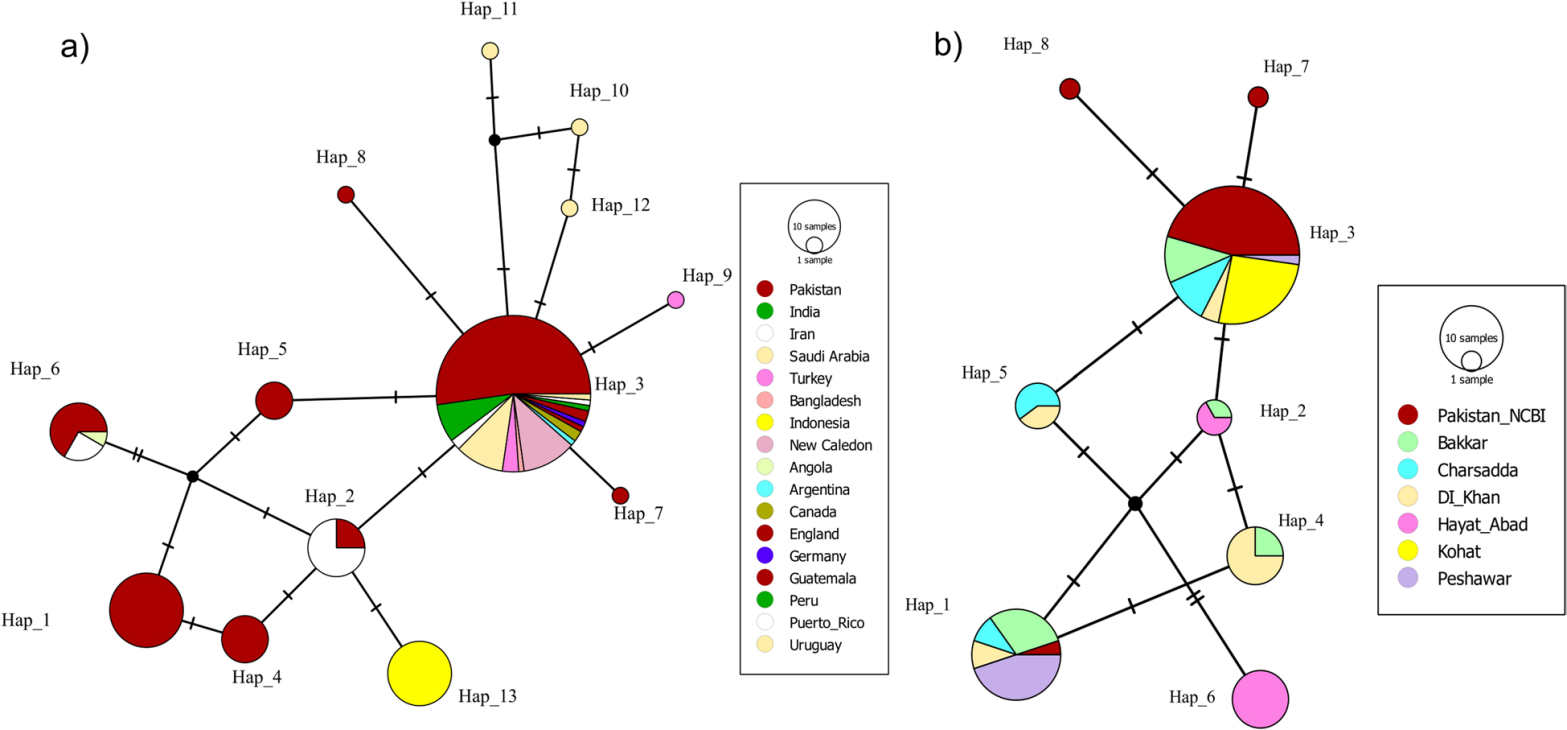
Global and regional haplotype networks based on mitochondrial *COI* sequences. **a** Global TCS network showing haplotypes from 20 countries. **b** Regional TCS network showing haplotypes from Pakistan, including GenBank (Pakistan_NCBI) and six sampling sites. Circle sizes are proportional to haplotype frequency. Colors indicate geographic origin. Hatch marks represent mutational steps; black nodes denote median vectors

The global haplotype network revealed a compact and shallow genealogy (Fig. 4a), with short mutational distances connecting haplotypes from Asia (Pakistan, India, Iran), the Middle East (Saudi Arabia, Turkey), and the Americas (e.g., Argentina, Guatemala, Canada). Hap_3 occupied the central position and was shared across multiple continents. Peripheral haplotypes, including those from Uruguay, Bangladesh, and Peru, were separated from the central node by one to four mutational steps. Several median vectors were present in the network, representing hypothetical ancestral or unsampled haplotypes.

A total of 13 distinct *COI* haplotypes (Hap_1 to Hap_13) were identified globally (Table 2). Pakistan revealed a higher number of haplotypes (*n* = 8), followed by Saudi Arabia (*n* = 4) and Iran (*n* = 3). Notably, the ten sequences from New Caledonia reported only one haplotype. Hap_3 was the most dominant, present in 53% of sequences (*n* = 88), with a broad geographic distribution. Rare haplotypes (Hap_7 to Hap_12) were found in only one individual each (0.6%). Higher genetic diversity was observed in Pakistani sequences (*k* = 1.75 and *π* = 0.007), followed by Iran (*k* = 1.35 and *π* = 0.006) and Saudi Arabia (*k* = 0.77 and *π* = 0.003).

**Table 2:** Country-wise distribution and relative frequency of *COI* haplotypes detected in global *Ae *. *aegypti* population resulting from haplotype networking analysis

Haplotypes	Pakistan	India	Iran	Saudi Arabia	Turkey	Bangladesh	Indonesia	New Caledonia	Angola	Argentina	Canada	England	Germany	Guatemala	Peru	Puerto Rico	Uruguay	HaP^a^ (n^b^)	Frequency (%)^c^
Hap_1	20	–	–	–	–	–	–	–	–	–	–	–	–	–	–	–	–	20	12
Hap_2	3	–	9	–	–	–	–	–	–	–	–	–	–	–	–	–	–	12	7.2
Hap_3	46	7	2	9	3	1	–	10	–	1	2	1	1	2	1	1	1	88	53
Hap_4	8	–	–	–	–	–	–	–	–	–	–	–	–	–	–	–	–	8	4.8
Hap_5	5	–	–	–	–	–	–	–	–	–	–	–	–	–	–	–	–	5	3
Hap_6	8	–	3	–	–	–	–	–	1	–	–	–	–	–	–	–	–	12	7.2
Hap_7	1	–	–	–	–	–	–	–	–	–	–	–	–	–	–	–	–	1	0.6
Hap_8	1	–	–	–	–	–	–	–	–	–	–	–	–	–	–	–	–	1	0.6
Hap_9	–	–	–	–	1	–	–	–	–	–	–	–	–	–	–	–	–	1	0.6
Hap_10	–	–	–	1	–	–	–	–	–	–	–	–	–	–	–	–	–	1	0.6
Hap_11	–	–	–	1	–	–	–	–	–	–	–	–	–	–	–	–	–	1	0.6
Hap_12	–	–	–	1	–	–	–	–	–	–	–	–	–	–	–	–	–	1	0.6
Hap_13	–	–	–	–	–	–	15	–	–	–	–	–	–	–	–	–	–	15	9
Total	92	7	14	12	4	1	15	10	1	1	2	1	1	2	1	1	1	166	

^a^number of haplotypes

^b^individuals

^c^relative proportion per haplotype

Within Pakistan, the haplotype network also displayed a compact structure (Fig. 4b), dominated by a central haplotype shared across all six sampling locations. In total, eight haplotypes (Hap_1–Hap_8) were identified (Table 3). Hap_3 was again the most frequent (50%, *n* = 46), occurring in every locality, while Hap_1 (21.7%), Hap_4 and Hap_6 (8.7% each), and Hap_5 (5.4%) showed more localized distributions. A unique haplotype (Hap_6) was observed in Hayat Abad. Notably, all twelve sequences from Kohat revealed only a single haplotype (Hap_3), with no intra-population variation observed. This limited diversity may reflect the small sample size (*n* = 12), as previous work indicates that at least 25–30 individuals are required to detect alleles at moderate frequencies (≥ 5%) [43]. The highest nucleotide diversity values were recorded in Bakkar (*k* = 1.54, *π* = 0.0063) and DI Khan (*k* = 1.45, *π* = 0.0059), while Kohat and Peshawar exhibited the lowest levels (Tables 4 and 5).

**Table 3:** Distribution and frequency of *COI* haplotypes in Pakistani *Ae *. *aegypti* population resulting from haplotype networking analysis

Haplotype	Pakistan_NCBI	Bakkar	Charsadda	DI Khan	Hayat Abad	Kohat	Peshawar	Hap (*n*)	Frequency (%)
Hap_1	1	6	2	2	–	–	9	20	21.7
Hap_2	–	1	–	–	2	–	–	3	3.3
Hap_3	21	5	5	2	–	12	1	46	50.0
Hap_4	–	2	–	6	–	–	–	8	8.7
Hap_5	–	–	3	2	–	–	–	5	5.4
Hap_6	–	–	–	–	8	–	–	8	8.7
Hap_7	1	–	–	–	–	–	–	1	1.1
Hap_8	1	–	–	–	–	–	–	1	1.1
Total	24	14	10	12	10	12	10	92

**Table 4:** Global genetic diversity estimates of *Aedes aegypti*

Estimate	Pakistan	India	Iran	Saudi Arabia	Turkey	Indonesia	New Caledonia	Canada	Guatemala
*n*^a^	92	7	14	12	4	15	10	2	2
s^b^	7	0	4	3	1	0	0	0	0
h^c^	8	1	3	4	2	1	1	1	1
Hd^d^	0.69	0	0.56	0.45	0.5	0	0	0	0
π^e^	0.007	0	0.005	0.003	0.002	0	0	0	0
k^f^	1.75155	0	1.35	0.77	0.50	0	0	0	0

^a^Number of sequences

^b^Number of variable/segregating sites

^c^Number of Haplotypes

^d^Haplotype (gene) diversity

^e^Nucleotide diversity (per site)

^f^Average number of nucleotide differences between two randomly chosen sequences from within the population

**Table 5:** Population-level genetic diversity estimates of *Aedes aegypti* from Pakistan

Estimate	Pakistan_NCBI	Bakkar	Charsadda	DI Khan	Hayat Abad	Kohat	Peshawar
*n*	24	14	10	12	10	12	10
s	5	3	3	3	3	0	3
h	4	4	3	4	2	1	2
Hd	0.24	0.71	0.69	0.73	0.36	0	0.2
π	0.002	0.006	0.005	0.006	0.004	0	0.0024
k	0.42	1.55	1.27	1.45	1.067	0	0.6

### Genetic diversity and population structure

Analysis of 166 sequences revealed notable geographic variability in genetic diversity (Tables 4 and 6). Pakistan exhibited the highest diversity (*n* = 92; *S* = 7; *h* = 8; n:h ratio = 13; Hd 0.69; *π* = 0.007), while populations from India, Indonesia, New Caledonia, and Canada displayed no detectable variation (Hd 0, *π* = 0), suggesting single-haplotype representation. Intermediate diversity was observed in Iran (Hd 0.56, *π* = 0.005), Saudi Arabia (Hd 0.45, *π* = 0.003), and Turkey (Hd 0.50, *π* = 0.002). The pooled global dataset yielded moderate haplotype diversity (Hd 0.70 ± 0.00125) and nucleotide diversity (*π* = 0.007). Neutrality tests revealed negative Tajima’s D (−0.547) and Fu’s Fs (−3.07), though both were insignificant (*P* > 0.10). Genetic differentiation was high (GST 0.3), with an estimated 29.5% of total variation explained by differences among populations. Correspondingly, gene flow was limited (Nm 1.19), reflecting restricted connectivity among geographically separated populations.

**Table 6:** Comparative summary of genetic diversity, neutrality, and within‑dataset population differentiation for the global and Pakistani *Ae*. *aegypti* datasets. FST values reflect pairwise differentiation among populations within each dataset

Estimate		Global population	Pakistani population
Genetic diversity^a^	L	715	692
	*n*	166	92
	S	12	7
	h	13	8
	Hd	0.70	0.69
	K	1.67	1.75
	π	0.007	0.007
	η	12	7
	R	9.7	8.5
Neutrality tests^b^	Tajima’s D	−0.54	0.65
	Fu’s Fs	−3.07	−0.076
Genetic differentiation^c^	GST	0.30	0.42
	Nm	1.19	0.7
	Fst	0.17	0.26

^a^L: length of region/number of sites (#nt), *n*: number of sequences, S: number of variable/segregating sites, h: number of haplotypes, Hd: haplotype (gene) diversity, K: average number of nucleotide differences between two randomly chosen sequences from within the population. π: nucleotide diversity (per site), η (Eta): total number of mutations, R: recombination estimator *R* = 4*Nr* where *N* is the population size and *r* is the recombination rate per sequence

^b^Tajima’s D: relative difference between observed (*π*) and expected genetic diversity, Fu’s Fs: frequency of haplotype (gene) distribution and (*k*)

^c^GST: inter-population genetic differentiation index. Nm: gene flow and population migration among populations; if diploid: Nm (1-FST)/4FST. FST: Wright’s F-statistics, pairwise genetic distance as a genetic differentiation index; under simple island model FST ≈ 1/(4Nm + 1)

Within Pakistan, 92 sequences from seven datasets exhibited regional heterogeneity in genetic diversity (Tables 5 and 6). Highest haplotype diversity was observed in DI Khan (Hd 0.73), Bakkar (Hd 0.71), and Charsadda (Hd 0.69). Kohat showed no diversity (Hd 0, *π* = 0), with all samples belonging to a single haplotype. Bakkar (*π* = 0.006) and DI Khan (*π* = 0.005) had the highest nucleotide diversity, whereas the archived Pakistani sequences from NCBI exhibited the lowest values for both haplotype diversity (Hd 0.24) and low nucleotide diversity (*π* = 0.002). Neutrality tests for Pakistani datasets (Tajima’s D = 0.652; Fu’s Fs = –0.076) were insignificant, consistent with neutral evolution in the *COI* region. Inter-district differentiation was high (GST 0.417; FST 0.26) and higher than the global estimate (FST 0.17) (Table 6), with intermediate gene flow (Nm 0.7), following Wright’s qualitative scale [44].

### Spatial genetic structure and differentiation among Pakistani populations

**Fig. 5:**
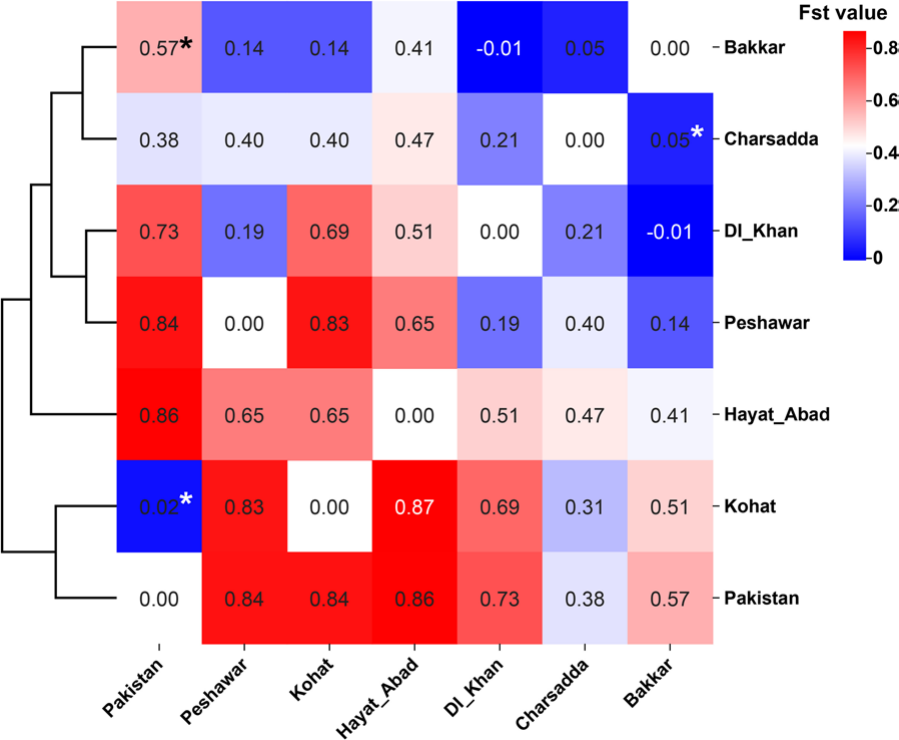
Pairwise FST heatmap and dendrogram of *Ae. aegypti* populations in Pakistan based on COI sequences. Color gradient shown from blue (low FST) to red (high FST) with white zero value cells representing the meeting point of each geographical region. Columns were clustered using Euclidean distance and complete linkage. The asterisk (*) represents insignificant *P*-value (> 0.05) as calculated by AMOVA

Pairwise FST values revealed variable levels of genetic differentiation among *Ae. aegypti* populations (Fig. 5). Estimates ranged from 0.01 between Bakkar and DI Khan to 0.87 between Kohat and Hayat Abad. The Pakistan_NCBI dataset was strongly differentiated from all other populations (FST 0.38–0.86). Kohat also showed high differentiation from most populations (FST 0.31–0.87), except with Pakistan_NCBI, where differentiation was negligible (FST 0.02).

Clustering analysis indicated that Kohat grouped with Pakistan_NCBI, Hayat Abad formed a distinct population, and the remaining populations grouped into one major cluster, albeit with internal differentiation. Most populations were highly differentiated, with the exception of Bakkar and DI Khan, which displayed low or even negative pairwise FST values.

AMOVA confirmed these patterns, attributing 41.4% of total variation to differences among populations within groups, 39.7% to intra-population variance, and 18.8% to differences between archived and new datasets (Table 7). Population differentiation among Pakistani districts was high based on pairwise FST (0.26; Table 6). By contrast, hierarchical AMOVA (grouping Pakistan_NCBI versus new sequences) yielded a very high overall fixation index (FST 0.603, *P* < 0.001), indicating strong subdivision when variance was partitioned among groups, among populations within groups, and within populations.

**Table 7:** AMOVA summary statistics for *Ae. aegypti* populations in Pakistan (groups: Pakistan_NCBI versus newly generated sequences) based on *COI* sequences

Component	Variance components (%)	Fixation index	Value
Among groups	0.68290 (18.83)	FST	0.60273
Among populations within groups	1.50282 (41.44)	FSC	0.51056
Within populations	1.44063 (39.73)	FCT	0.18832

### Detection of *Wolbachia* in wild *Ae. aegypti* populations

None of the mosquito specimens (*n* = 300) tested positive for natural *Wolbachia* infection. The absence of amplification in all field samples, despite successful amplification of internal control markers confirming DNA integrity and PCR efficiency, suggests that *Wolbachia* is either absent or present below detectable thresholds. To validate assay performance, DNA from *Ae. albopictus* and *Culex pipiens* (known *Wolbachia*-positive species) was used as positive controls. These controls consistently yielded qPCR amplification with Cq values ranging between 17.5 and 21.0, indicating robust detection sensitivity and assay functionality. Negative controls (nuclease-free water) showed no amplification. Collectively, these results support the absence of natural *Wolbachia* infection in wild *Ae. aegypti* populations in Pakistan, consistent with [21].

## Discussion

This study provides the first integrated assessment of the genetic structure and *Wolbachia* infection status of *Ae. aegypti* in Pakistan. Pakistani populations exhibit moderate mitochondrial diversity and higher population genetic structure, cluster within a single global lineage, and show no evidence of natural *Wolbachia* infection. These findings provide a foundation for understanding vector ecology and planning future surveillance.

### Phylogenetic and phylogeographic context

Analysis of 166 *COI* sequences confirmed that Pakistani *Ae. aegypti* form part of a monophyletic global lineage, with very high sequence similarity (> 99.6%). This pattern indicates a recent common ancestry consistent with colonization events, while ecological fragmentation within Pakistan may have contributed to localized genetic differentiation. Similar signatures of high mitochondrial homogeneity with regional structuring have been reported in Central America and Asia [11, 45]. Notably, Pakistani sequences clustered with regional neighbors (Saudi Arabia, Iran, and India), reflecting homogeneity (Fig. 2). By contrast, geographically distant populations (South America and Oceania) showed deeper divergence, aligning with global phylogeographic trends. Compared with ancestral African populations [46], most Asian *Ae. aegypti* showed reduced mitochondrial diversity, reflected in phylogenetic intermixing. However, Pakistan exhibited relatively higher diversity than neighboring countries (Table 2), suggesting rapid population growth after a bottleneck event reflected by high Hd and low nucleotide diversity (π).

### Haplotype diversity and intra-country differentiation

These phylogenetic insights are further supported by haplotype-based analyses, which reveal patterns of diversity and differentiation within and across populations. *COI* sequence analysis identified 13 global haplotypes, including eight detected within Pakistan. Haplotype 3 was the most prevalent globally (50–53%) and within Pakistan, mirroring patterns reported from Southeast Asia, Australasia [3], and El Salvador [11]. By contrast, neighboring regions such as Iran (two haplotypes) [13] and India (five haplotypes) [47] reported lower diversity, although comparisons must be made cautiously given differences in sample size. Large datasets (e.g., India, *n* = 589) detect more rare variants, while smaller datasets risk underestimating diversity [43, 48]. Our sample size (*n* = 92) was sufficient to detect multiple haplotypes but may still underestimate diversity in under-sampled sites.

Within Pakistan, the Kohat and Hayat Abad populations showed strong differentiation (strong FST values; Fig. 5) compared with other sites’ populations. The haplotype network supports the observed genetic differentiation; 80% (8/10) of haplotype-6 sequences originated from Hayat Abad, suggesting localized genetic homogeneity (Table 3). By contrast, Kohat differed from most populations but shared continuity with archived sequences, potentially reflecting sampling bias due to small sample size (*n* = 12), an observation similar to [41]. The network’s bush-like structure, dominated by Hap_3, suggests shallow differentiation consistent with human-mediated dispersal. These patterns mirror findings from South Asia and the Middle East [7, 8, 48] and the Indo-Pacific and Pacific [43–45], where urbanization mobility promotes gene flow in *Ae. aegypti,* yet localized ecological or geographic barriers may maintain differentiation [49].

### Population differentiation and gene flow

Globally, *Ae. aegypti* populations showed high genetic differentiation (FST 0.17, GST 0.30) and neutrality (insignificant Tajima’s D and Fu’s Fs; Table 6). Pakistani populations, however, showed very high genetic differentiation (FST 0.26; GST 0.42), intermediate gene flow (Nm 0.71), and evidence of low dispersal (Tables 6 and 7). Because COI is maternally inherited and haploid, migration estimates derived from FST rest on strong assumptions; we therefore interpret Nm cautiously and emphasize FST/AMOVA as more reliable indicators of structure. AMOVA attributed > 40% of variation to intra-population differences, while UPGMA revealed three distinct clusters, suggesting ecological gradients or microclimate barriers contribute to structure. These findings are consistent with Iranian and Indian studies linking fragmentation to urban landscape and control interventions [13, 49]. Although *COI* markers offer limited resolution for fine-scale inference [3], our results highlight meaningful differentiation that should be validated with nuclear or genomic markers. Genetically distinct populations may differ in vector competence, insecticide resistance, intervention response, or susceptibility to *Wolbachia* invasion [34, 50], reinforcing the need for geographically tailored strategies [3, 51].

### *Wolbachia* detection and vector control implications

All field-collected *Ae. aegypti* tested negative for *Wolbachia,* consistent with large-scale surveys showing a global absence of natural *Wolbachia* in *Ae. aegypti* (2663 wild-caught specimens across 27 countries) [21], but contrasting with sporadic low-prevalence reports from the Philippines, Malaysia, India, and the USA [22–27, 52], Methodological variations, including sample pooling and marker sensitivity, may explain discordance [52], as false positives can result from nematode-derived *Wolbachia* (e.g., *Dirofilaria immitis*) [52, 53]. Our rigorous individual-level screening, optimized PCR protocols, and validated controls (including *Wolbachia* detection in *Ae. albopictus* and *Culex spp*.; unpublished data) ensure result reliability. Although highly divergent or low-density *Wolbachia* strains cannot be entirely ruled out, our findings indicate that natural infections are either absent or occur at levels below current detection limits in these populations.

The absence of natural *Wolbachia* infections in Pakistani *Ae. aegypti* provides a critical biological baseline for both ecological inference and vector control applications. Globally, *Wolbachia*-based interventions have reduced dengue incidence by −77% in Yogyakarta, Indonesia [54], and 57% in Singapore [55]. Importantly, the lack of natural *Wolbachia* infections in Pakistan eliminates compatibility barriers, increasing the likelihood of successful establishment of introduced strains [56]. Recent experimental work in Pakistan has already demonstrated stable transinfection of *Ae. aegypti* with *w*AlbB, with high maternal transmission fidelity and minimal fitness costs under semi-field conditions [57], further underscoring the feasibility of such interventions. These findings directly inform Pakistan’s dengue control programs, where *Wolbachia*-based biocontrol is emerging as a viable complement to conventional interventions. By integrating *Wolbachia* screening with COI sequences analyses, our study also addresses a key methodological concern. In *Ae. albopictus* and other insects, *Wolbachia*-driven mitochondrial sweeps have reduced haplotype diversity and biased phylogeographic inference [15–18]. Confirming *Wolbachia* absence in *Ae. aegypti* populations in Pakistan ensures that observed COI diversity reflects host demographic and evolutionary processes rather than endosymbiont effects. This dual approach strengthens the rigor of our findings and provides essential insights for future *Wolbachia*-based dengue control strategies tailored to Pakistan.

### Limitations and future directions

Several limitations should be acknowledged. First, reliance on a single mitochondrial marker constrains resolution; nuclear markers or whole-genome sequencing will provide more robust insights into demography, adaptation, and gene flow. Second, although sampling was conducted across ecologically diverse regions, sample sizes at several locations were relatively small. Limited sampling (< 30) reduces the power to detect low-frequency haplotypes and may underestimate true genetic diversity [14, 41]. Third, inclusion of archived GenBank sequences introduced heterogeneity in metadata and sequencing quality, potentially biasing comparisons. Future studies should aim for a balanced, standardized sampling across sites and ensure metadata quality when incorporating external sequences. Lastly, the data were collected during a single mosquito season (July–December 2021), limiting our ability to evaluate seasonal or interannual trends in genetic structure and *Wolbachia* prevalence. To validate and extend these findings, future studies should incorporate larger, temporally replicated samples analyzed with genome-wide sequencing (e.g., next-generation sequencing) and employ highly sensitive *Wolbachia* detection methods such as digital PCR.

## Conclusions

Our findings demonstrate that *Ae. aegypti* populations globally form a phylogenetically monophyletic lineage with high genetic differentiation. Pakistani populations are likewise monophyletic, with moderate mitochondrial diversity, intra-country heterogeneity, and very high population structure, and they cluster closely with neighboring countries. Although spatial structuring was evident, confirmation with nuclear markers is needed to resolve potential differentiation and its epidemiological relevance. The absence of detected *Wolbachia* infections suggests that natural infections are either absent or occur at very low prevalence. Continued genomic surveillance and broader sampling are recommended to guide vector control strategies.

## Supplementary Information

Supplementary Material 1: Table S1. Country-wise distribution and GenBank accession numbers of *Ae. aegypti COI* sequences used in this study. This table includes 166 COI sequences: 68 newly generated and 98 retrieved from GenBank.

## Data Availability

All mitochondrial *COI* sequences generated in this study have been deposited in GenBank under accession numbers PV925869–PV925936. Other supporting datasets analyzed during this study are available from the corresponding author upon reasonable request.
