# Publisher Correction: Population genetic diversity and natural *Wolbachia* infection in *Aedes aegypti* from Pakistan

**DOI:** 10.1186/s13071-025-07196-x

**Published:** 2026-01-05

**Authors:** Jehangir Khan, Datao Lin, Amer Al-Jawabreh, Abdul Aziz, Dongjing Zhang, Tao Chen, Qian Han

**Affiliations:** 1 Laboratory of Tropical Veterinary Medicine and Vector Biology, School of Life and Health Sciences, Hainan Province Key Laboratory of One Health, Collaborative Innovation Center of Life and Health, Hainan University, Haikou, 570228 Hainan China; 2 Hainan General Hospital, Hainan Affiliated Hospital of Hainan Medical University, Haikou, 570100 Hainan China; 3 Zoology Department, Abdul Wali Khan University, Mardan, Pakistan; 4 Chinese Atomic Energy Agency Center of Excellence On Nuclear Technology Applications for Insect Control, Key Laboratory of Tropical Disease Control of the Ministry of Education, Sun Yat-Sen University, Guangzhou, China; 5 Department of Parasitology, Key Laboratory of Tropical Disease Control (Ministry of Education), Zhongshan School of Medicine, Sun Yat-Sen University, Guangzhou, China; 6 Department of Medical Laboratory Sciences, Faculty of Allied Health Sciences, Arab American University, Jenin, Palestine; 7 Leishmaniases Research Unit, Jericho, Palestine; 8 Nuclear Institute for Food and Agriculture (NIFA), Pakhtunkhwa, Pakistan; 9 Hainan Provincial Bueau of Disease Prevention and Control, Haikou, 570100 China

**Publisher Correction: Parasites & Vectors (2025) 18:486** 10.1186/s13071-025-07134-x

Following publication of the article, it was brought to the journal's attention that because of an error during production of the article for publication, the author list was incorrectly ordered. In addition, the following grant was missing from the Funding information of the article: The National Natural Science Foundation of China (U22A20363). The authorship list and the Funding have now been corrected, and the correct authorship list is reflected in this erratum [1]. The publisher thanks you for reading this erratum and apologizes for any inconvenience caused.
